# Advancing Proton Therapy: Integration of Minibeam Spatial Fractionation and FLASH Dose Rates

**DOI:** 10.21203/rs.3.rs-8116270/v1

**Published:** 2025-12-01

**Authors:** yuting lin, Wei Wu, Jufri Setianegara, Aoxiang Wang, Nicolas Gerard, Jarrick Nys, Gregory Gan, Hao Gao

**Affiliations:** The University of Texas Southwestern Medical Center; Chinese Academy of Sciences; University of Pennsylvaniaa; University of Kansas Medical Center; Ion Beam Applications; Ion Beam Applications; University of Kansas Medical Center; The University of Texas Southwestern Medical Center

**Keywords:** Spatially Fractioned Radiation Therapy, FLASH, Minibeam, Pencil Beam Scanning

## Abstract

**Background:**

Proton minibeam radiation therapy (pMBRT) introduces spatial fractionation of dose distributions at submillimeter resolution, offering a promising approach to reducing normal tissue toxicity while maintaining effective tumor control. However, the high monitor unit (MU) requirements of multi-slit collimators (MSC) result in extended delivery times, posing a significant challenge. This study explores the feasibility of integrating pMBRT with ultra-high-dose-rates (UHDR) to overcome this limitation while leveraging potential biological synergies to enhance the therapeutic index and advance clinical applications.

**Methods:**

The study utilized the IBA Proteus^®^ONE compact proton therapy system equipped with two MSCs, each with center-to-center distances of 2.8 mm, slit widths of 1.0 mm, and thicknesses of 6.5 cm and 10 cm. FLASH delivery was achieved with 228 MeV protons at a current of 125 nA, while clinical beams operated at 226 MeV with 1–5 nA. Dose measurements using Gafchromic films in solid water phantoms were compared with Monte Carlo simulations. Delivery times were compared for FLASH and clinical beams. PBS dose rate was calculated based on the spot delivery log file.

**Results:**

The study successfully demonstrated pMBRT dose distributions under FLASH dose rates, significantly reducing treatment times to 2.5 seconds compared to 3 minutes for clinical beams. The 10 cm collimator achieved higher peak-to-valley dose ratios (PVDRs) at 2 cm depth (4.36) than the 6.5 cm collimator (2.57), optimizing FLASH delivery conditions. Results highlight the potential to improve dose delivery efficiency while maintaining spatial resolution and dose modulation, supporting future clinical advancements.

**Conclusion:**

This study demonstrates the feasibility of integrating pMBRT with FLASH dose rates using a clinical proton therapy system. By addressing challenges associated with delivery times and leveraging the combined advantages of spatial fractionation and ultra-high-dose-rates, this work paves the way for the clinical translation of pMBRT with FLASH, offering innovative possibilities for treating challenging malignancies with high-dose precision therapy.

## 1. Introduction

Spatially fractionated radiation therapy (SFRT) differs from conventional radiation therapy by using highly modulated dose distributions across microscopic or submillimeter spatial scales. to enhance the therapeutic index of radiation treatments ^1–4^. Among various SFRT techniques, minibeam radiation therapy (MBRT) has attracted growing interest over the past decade due to its ability to deliver highly heterogenous dose across to small tumor volume, overcoming the traditional use of SFRT on bulky unresectable hard to treat tumors ^5–11^. When implemented with protons, also known as proton minibeam radiation therapy (pMBRT), combines the spatially fractionated advantage of MBRT with the advantage of proton beam with no exit dose ^6,12,13^. In addition to physical advantages, emerging overwhelming preclinical data suggest that tumor biology may respond differently to pMBRT invoking distinct immune-modulatory pathways that could enhance therapeutic outcomes ^14–16^.

Despite these promising features, the delivery of pMBRT remains challenging. Its implementation rely on metallic multi-slit collimators (MSCs) to spatially modulate the proton field to produce alternating high- and low-dose regions. However, these devices inherently block a substantial fraction of the primary beam, resulting in high monitor unit (MU) requirements and prolonged delivery times ^17–19^. Extended irradiation times not only cause patient discomfort, but also increase susceptibility to motion-induced blurring, which can degrade the desired minibeam pattern and reduce peak-to-valley dose ratio (PVDR).

In parallel, the emergence of ultra-high-dose-rate (UHDR) irradiation, known to trigger the FLASH effect, has introduced a transformative concept in radiotherapy ^1,20,21^. FLASH irradiation, delivered at dose rates exceeding approximately 40 Gy/s, has been shown in numerous preclinical studies to spare normal tissues while maintaining tumor control. Proton beams are also particularly well-suited for FLASH delivery, as modern accelerator systems can achieve UHDR conditions with appropriate tuning of beam current and pulse structure ^21–25^.

The combination of pMBRT and FLASH irradiation represents a compelling new direction in radiation therapy research ^1,10,18,26^. While the underlying biological mechanisms of spatial fractionation and ultra-high-dose-rate effects are not yet fully understood, their integration offers both biophysical and practical advantages. From a technical standpoint, operating at UHDR-level beam currents can dramatically reduce pMBRT delivery time, mitigating the limitations imposed by MSC-based beam blocking and improving temporal stability against patient motion. Furthermore, the potential synergistic biological effects—combining spatial and temporal sparing mechanisms—may further enhance the therapeutic ratio.

In this work, we present the first demonstration of integrating pMBRT with FLASH-dose-rate delivery on a clinical compact proton therapy system. We achieved submillimeter beamlet modulation and verified the resultant dose distributions with Gafchromic film measurements and Monte Carlo simulations. We further evaluated the corresponding delivery times, dose-rate characteristics, and peak-to-valley dose ratios under FLASH and conventional conditions. This study establishes the technical feasibility of proton minibeam FLASH therapy, paving the way toward its clinical translation and potential application for challenging malignancies requiring high-dose precision with improved normal tissue sparing.

## 2. Material and Method

### 2.1. Proton radiation unit in conventional and FLASH mode

The pMBRT system used in this study has been described previously ^12,17,27–29^. The experiments were performed using a single-room PBS proton machine (IBA Proteus^®^ONE, Louvain-La-Neuve, Belgium). The commissioned clinical system is capable of delivering proton energies up to 226 MeV. The PBS delivery system utilizes two orthogonal scanning magnets to position the proton pencil beam at the desired position. Due to the geometric design of the scanning system, the source-to-axis distances (SADs) differ between the two scanning directions. The SAD was measured to be 298 cm in the X-direction (patient’s right–left direction) and 970 cm in the Y-direction (patient’s superior–inferior direction). This geometric asymmetry is intrinsic to gantry design and has been accounted for in all dosimetric and Monte Carlo modeling procedures.

For conventional clinical delivery, the beam current at isocenter, typically ranged from 0.1 to 1 nA, corresponding to a dose rate in the range of 0.05–0.5 Gy/s, depending on field size and beam energy. The FLASH mode delivery was achieved on the same system through a series of beamline optimizations that enhanced transmission efficiency and increased extracted current. Specifically, beam transport optics were re-tuned to minimize beam losses, and spot size parameters were adjusted to maintain focusing performance at elevated current levels.

In the FLASH configuration, the delivery time structure remains the same, the S2C2 operated at its nominal 1 kHz pulse repetition rate with a fixed pulse width of approximately 10 μs ^28^. Under these conditions, a beam current of up to 125 nA at isocenter was measured using the Faraday cup ^28,30^. The total charge per pulse was also characterized to verify linearity and stability across repeated measurements. These beam delivery parameters were subsequently used to compare pMBRT delivery efficiency between conventional and FLASH modes.

It is noteworthy that the manufacturer has outlined a future roadmap to increase the extractable beam current of the S2C2, which would further expand the range of achievable UHDR conditions and facilitate even faster pMBRT delivery.

### 2.2. Minibeam multi-slit collimator (MSC)

**Figure 1:**
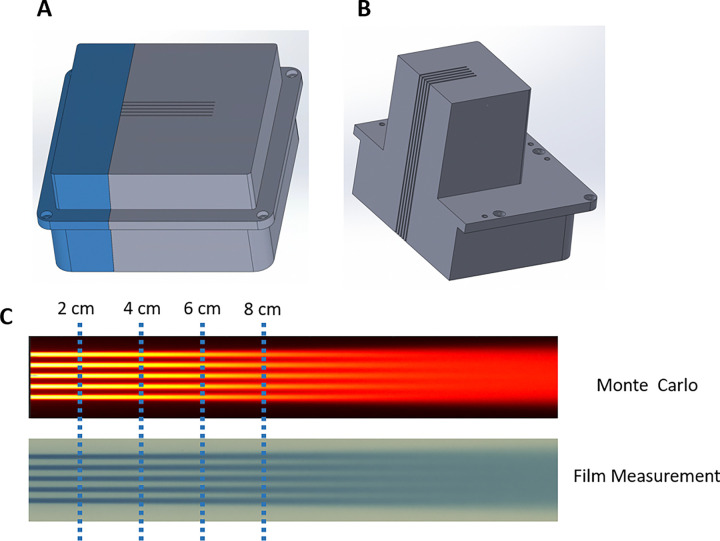
Proton minibeam multi-slit collimators and depth-dose validation. (A) 6.5 cm-thick collimator and (B) 10.0 cm-thick collimator, each featuring five slits with 1.0 mm width and 2.8 mm center-to-center spacing. Both collimators were fabricated from brass and mounted on the small snout of the Proteus^®^ONE nozzle. (C) Comparison of percent-depth-dose (PDD) curves between Monte Carlo simulation and Gafchromic film measurements. The depths corresponding to the laterally extracted profiles used for quantitative analysis are indicated.

The proton minibeam collimator (MSC) was mounted on the small snout that was securely latched to the distal end of the nozzle, functioning as a beam-modifying accessory, serving as a beam-modifying accessory. Two collimators were evaluated under the UHDR proton beam. Each collimator comprised five parallel slits with a center-to-center spacing of 2.8 mm and an individual slit width of 1.0 mm, Fig. 1A and 1B.

The slit array was oriented along the X-axis, while the beam divergence occurred primarily in the Y-direction (source-to-axis distance = 910 cm). Both collimators were machined using brass and custom-designed and manufactured by DotDecimal, Inc. (.decimal^®^, Sanford, Florida, USA).

### 2.3. Experimental validation

FLASH delivery was performed using 228 MeV protons at a beam current of 125 nA, in contrast to conventional clinical beams operated at 0.1–1 nA. Dosimetric characterization measurements included Percentage depth-dose (PDD) and lateral dose profiles using Gafchromic films embedded in solid water phantoms, shown in Fig. 1C. The procedure for film-based PDD measurement has been described in detail previously^12,17,27^. The irradiation field was configured as a 3 cm × 3 cm square field, delivered via a raster-scanned pattern comprising 49 spots with 5 mm center-to-center spacing.

Film calibration was performed using a 226 MeV clinical beam at a depth of 2 cm in solid water (Ashland, Bridgewater, NJ, USA). After irradiation, the films were digitized using an Epson Expression 11000XL flatbed scanner (Epson America, Inc., Los Alamitos, CA, USA) at 300 dpi resolution, corresponding to a pixel spacing of 0.085 mm. Dose conversion and quantitative analysis were conducted using the IBA myQA Film software package (IBA Dosimetry, Schwarzenbruck, Germany). Film response calibration curves were applied consistently for both reference and UHDR conditions to ensure relative accuracy across the measured datasets.

### 2.4. Monte Carlo simulation

The Monte Carlo (MC) simulation for the study is performed using TOPAS and employs a water phantom with dimensions of 10×10×40 cm^3 17,31^. The phantom volume is discretized into a non-uniform grid structure with spatial resolution of 1 mm in both x and z directions, while maintaining a 0.1 mm resolution in y direction to capture fine lateral minibeam dose variations induced by the multi-slit collimator. The following Geant4 physics lists were applied: *g4em-standard_opt4*, *g4h-phy_QGSP_BIC_HP*, *g4decay*, *g4ion-binarycascade*, *g4h-elastic_HP*, and *g4stopping*, while all other parameters remained at default settings. In addition to the total dose, each individual pencil beam spot was simulated separately to allow post-processing and calculation of both cumulative dose distributions and instantaneous dose rate metrics.

### 2.5. Dose Rate Calculation

Two complementary approaches were employed for dose rate quantification: the total average dose rate and the pencil-beam scanning (PBS) dose rate. The former represents a macroscopic, field-averaged quantity, while the latter captures localized temporal dose dynamics at the voxel level ^22,32,33^.

#### 2.5.1 Total Average Dose Rate

The total average dose rate is defined as the ratio between the total dose delivered to a point and the total irradiation time for the entire field:

1 $$\dot{D}(x)=\frac{D(x)}{t_{\mathrm{total}}}$$

where D(x) is the cumulative dose at position x, and $t_{\text{total}}$ is the total beam-on time required for the field delivery. This definition represents the overall mean dose rate experienced by a voxel during the complete irradiation sequence, without accounting for potential local dose rate effects stemming from the proton PBS delivery.

#### 2.5.2 PBS dose rate

The PBS dose rate metric, originally proposed by Folkerts *et al.* accounts for the temporal characteristics of individual spot deliveries and local dose accumulation ^34^. It is expressed as:

2 $$\dot{D}(x{)}^{PBS}=\frac{D(x)\;-\;2D^{th}}{t_{1,i}\;-\;t_{0,i}}$$

where $t_{1,i}=t_{i}\left(D_{i}-D^{\mathrm{th}}\right)$ and $t_{0,i}=t_{i}\left(D^{\mathrm{th}}\right)$, which are the delivery times to deliver a cumulative dose of $D_{i}-D^{\mathrm{th}}$ and $D^{\mathrm{th}}$ of the i^th^ voxel respectively. $D_{i}$ refers to the total dose received by the i^th^ voxel and $D^{\mathrm{th}}$refers to the PBS threshold dose.

As seen, the PBS dose rate captures potential FLASH sparing arising from local dose rate effects due to proton PBS delivery. Embedded within the definition is the hypothesis whereby the bulk of the biological FLASH sparing experienced by the i^th^ voxel is controlled by the local delivery of $D_{i}-D^{\mathrm{th}}$ dose, discounting the elongated time to deliver the initial and final $D^{\mathrm{th}}$ doses from neighboring spots. In this study, a threshold dose of 0.5 Gy was used for PBS dose rate calculations. Currently, there isn’t a clear consensus in scientific literature on an appropriate level and hence, this value was selected as an initial approximation which represents a clinically insignificant dose level relative to the magnitude of the therapeutic doses delivered in this work. We also verified that the choice of the threshold dose did not greatly impact the PBS dose rate calculations.

## 3. Results

### 3.1 Film Measurements and Monte Carlo Validation

**Figure 2:**
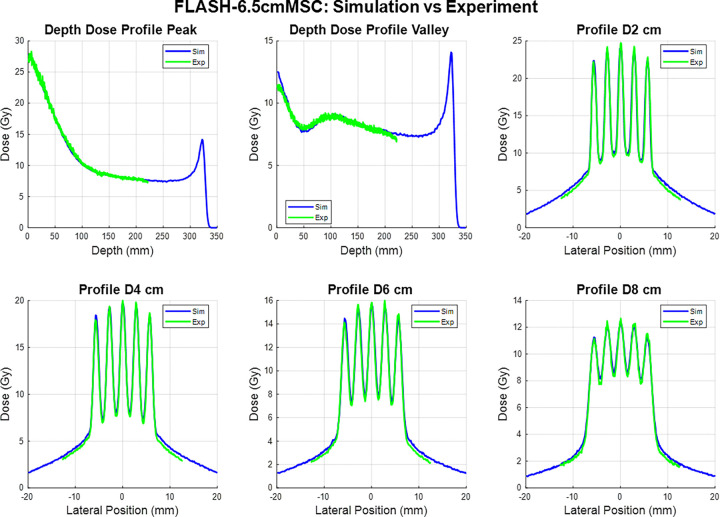
Comparison between simulated (blue) and measured (green) dose distributions obtained under 228 MeV FLASH beam conditions for 6.5 cm thick MSC. The top row shows (left) depth-dose profiles through the beam peak and (middle) valley regions, and (right) the lateral dose profile at 2 cm depth. The bottom row shows lateral dose profiles at depths of 4 cm, 6 cm, and 8 cm, respectively. Simulated and experimental results exhibit excellent agreement in both peak position and valley modulation, confirming accurate modeling of the multi-slit geometry and beam divergence in the Monte Carlo simulation.

**Figure 3:**
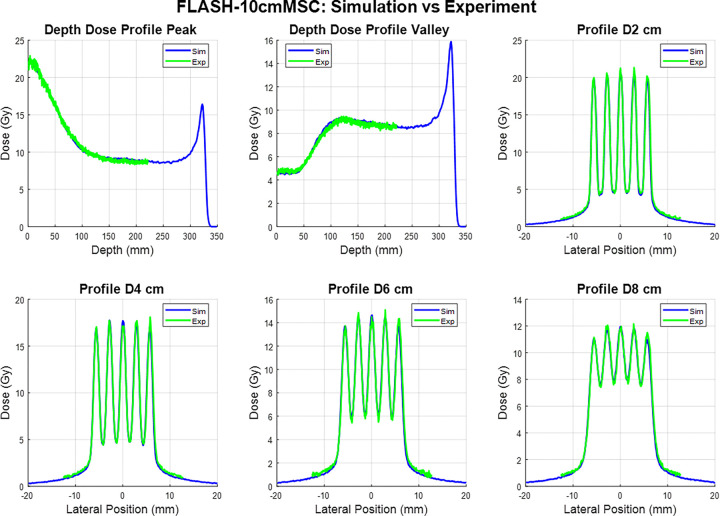
Comparison between simulated (blue) and measured (green) dose distributions obtained under 228 MeV FLASH beam conditions for 10.0 cm thick MSC. The top row shows (left) depth-dose profiles through the beam peak and (middle) valley regions, and (right) the lateral dose profile at 2 cm depth. The bottom row shows lateral dose profiles at depths of 4 cm, 6 cm, and 8 cm, respectively. Simulated and experimental results exhibit excellent agreement in both peak position and valley modulation, confirming accurate modeling of the multi-slit geometry and beam divergence in the Monte Carlo simulation.

The experimental film measurements and corresponding Monte Carlo (MC) simulation results for the two proton minibeam multi-slit collimators (MSCs) under FLASH beam conditions are presented in Figs. 2 and 3. Each figure includes percent-depth-dose (PDD) curves for the peak and valley regions, as well as lateral dose profiles at depths of 2 cm, 4 cm, 6 cm, and 8 cm.

The measured film data and MC simulations demonstrate excellent agreement in both depth-dose and lateral profiles. The overall shape, relative peak positions, and modulation patterns of the minibeam structures are consistently reproduced by the simulations for both collimators. Across all evaluated depths, the periodicity and spatial modulation of the dose peaks are well aligned between simulation and experiment, confirming accurate modeling of the collimator geometry and beam transport through the slit array.

The 6.5 cm-thick collimator produced well-defined peak–valley structures that remained distinct up to approximately 8 cm depth, with slight smoothing of valley regions at deeper depths due to lateral scattering. The 10 cm-thick collimator exhibited sharper beam edges and enhanced peak-to-valley separation, attributable to its longer brass path length and improved lateral collimation. The result indicates that the MC simulation accurately reproduces both the spatial modulation and depth-dependent attenuation observed in film measurements.

Overall, these results demonstrate strong correspondence between experimental and simulated data, establishing confidence in the accuracy of the beamline model and validating its use for subsequent dose-rate and spatial-fractionation analyses.

### 3.2 PBS dose rate profile

**Figure 4:**
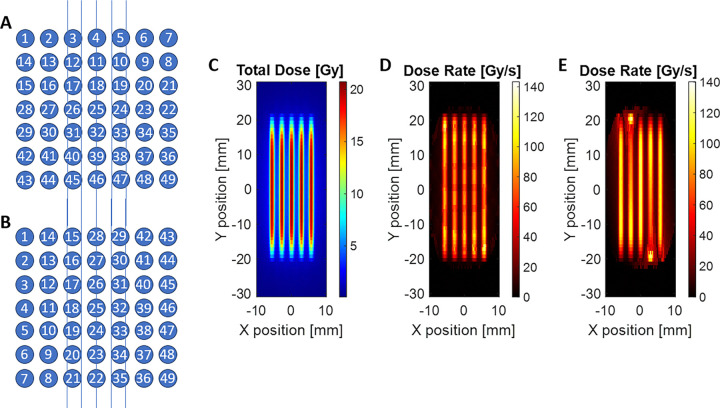
(A) and (B) Two possible raster-scanning sequences used to deliver a 3 × 3 cm^2^ proton minibeam field consisting of 49 spots. (C) Simulated total-dose distribution showing equivalent cumulative dose between the two scanning strategies. (D) and (E) Corresponding PBS dose-rate maps computed using Equation (2) according to Pattern A and B. Although the total dose is identical, the instantaneous dose-rate pattern varies depending on the scanning order, demonstrating the influence of spot delivery sequence on local FLASH dose-rate heterogeneity.

The PBS dose-rate distributions were calculated according to Eq. (2), using the simulated per-spot dose and timing information. Two raster-scanning patterns were evaluated, referred to as Pattern A and Pattern B, as shown in Figs. 4A and 4B. The actual delivery sequence for the results in 3.1 is Pattern A. Each pattern delivered a 3 × 3 cm^2^ field composed of 49 scanning spots with 5 mm center-to-center spacing. The difference between the two patterns lies in the spot delivery order, while all other irradiation parameters—beam current, energy, and total monitor units—were kept identical.

The resulting total dose distribution was equivalent for both patterns (Fig. 4C), confirming consistent spatial dose uniformity. However, the instantaneous PBS dose-rate maps (Figs. 4D–E) showed clear spatial variations depending on the scan order. The observations emphasize that, even under identical total-dose conditions, the temporal sequence of spot delivery can significantly influence the local dose-rate pattern in PBS FLASH delivery. Careful optimization of the scanning strategy is therefore essential to balance total-field homogeneity and maintenance of ultra-high-dose-rate conditions.

### 3.3 Delivery Time Comparison

A quantitative summary of the peak-to-valley dose ratio (PVDR) and dose-rate metrics for both conventional and FLASH beam deliveries is presented in Table 1. For the same 3 × 3 cm^2^ pMBRT field, the FLASH beam (228 MeV) achieved ultra-high-dose-rate (UHDR) delivery with an average beam current of 125 nA, resulting in a total delivery time of approximately 2.5 seconds—about 66 times faster than the conventional 226 MeV beam, which required 166 seconds for an equivalent field.

Under FLASH conditions, both the 6.5 cm and 10 cm collimators preserved comparable physical and dosimetric characteristics relative to conventional operation. The PVDR values measured at depths of 2 cm, 4 cm, and 6 cm remained consistent within experimental uncertainty, confirming that UHDR operation does not degrade spatial modulation or field uniformity.

The 10 cm collimator demonstrated systematically higher PVDRs than the 6.5 cm design, particularly at shallow depths (e.g., 4.37 ± 0.15 vs. 2.57 ± 0.15 at 2 cm), reflecting its improved lateral confinement of the proton minibeams. The corresponding peak dose rate (PDR) values exceeded 100 Gy/s under PBS-based dose-rate calculation (${\dot{D}}_{\mathrm{PBS}}$), fulfilling the threshold for FLASH effect induction.

These results collectively confirm that FLASH-pMBRT delivery can achieve clinically meaningful spatial fractionation and ultra-high-dose-rates simultaneously, without compromising beam quality or PVDR preservation.

## 4. Discussion

This work represents the first experimental demonstration of integrating proton minibeam radiation therapy (pMBRT) with ultra-high-dose-rate (FLASH) delivery on a clinical compact proton therapy system. While several review papers have previously proposed this combination as an “ideal synergy”—merging spatial and temporal normal-tissue sparing mechanisms—our study provides the first empirical evidence that both can be simultaneously realized using an existing clinical beamline ^1,10,18,35^. Prior to this, the concept of combining spatial fractionation with FLASH had been explored only through theoretical analyses and Monte Carlo simulations, and more recently, an electron FLASH simulation study demonstrated the potential feasibility of generating minibeam-like dose distributions under UHDR conditions ^10^. The present work advances the field from conceptual proposals to tangible proof of principle using a clinical proton system.

By achieving dose rates exceeding 100 Gy/s while maintaining submillimeter beam modulation and stable peak-to-valley dose ratios (PVDRs), this study demonstrates that UHDR pMBRT delivery is technically feasible without compromising spatial resolution. This finding confirms that the mechanical and dosimetric constraints often associated with multi-slit collimators can be effectively mitigated by operating in FLASH mode, reducing delivery times by more than 60-fold compared with conventional beams. The ability to deliver spatially fractionated dose distributions within a few seconds has important implications for motion mitigation, beam stability, and future in-vivo research, as it enables the full FLASH time structure to be preserved even in highly modulated fields.

A major limitation of the current implementation is the use of a single high-energy (228 MeV) beam in open-field geometry. While this configuration allowed the achievement of UHDR conditions, it does not yet reproduce the spread-out Bragg peak (SOBP) typically required for clinical depth coverage. However, with the recent progress in conformal or energy-layered FLASH techniques, the addition of the multi-slit collimator as an accessory at the nozzle could enable SOBP-based pMBRT under FLASH conditions ^21,36–38^. Such an approach would allow FLASH-pMBRT delivery with conformal dose coverage while retaining the lateral spatial fractionation benefit, and potentially benefit patients with jointly optimized FLASH, PVDR, and dose parameters^39–41^.

In this study, quantitative measurements were limited to total dose distributions, while the dose-rate evaluation was derived computationally from the machine’s delivery structure and log-file data. This approach provides a reliable estimate of instantaneous dose-rate behavior but does not capture real-time spatiotemporal dose-rate fluctuations within individual pulses or scan sequences. As the field advances, the development and integration of high-speed, high-dynamic-range two-dimensional detectors—capable of microsecond temporal resolution—will be crucial for experimentally validating dose-rate maps under UHDR conditions ^42,43^. Such detectors would greatly enhance the ability to characterize and optimize pMBRT and FLASH delivery, bridging the current gap between simulated dose-rate models and direct experimental verification.

The combination of spatial fractionation and UHDR irradiation represents an exciting paradigm shift in radiotherapy. Although physical feasibility has now been experimentally validated, the biological consequences of combining FLASH and pMBRT remain to be determined. Future animal studies will be essential to evaluate whether dual-modality delivery elicits additive or synergistic normal-tissue sparing effects. Preclinical investigations should focus on comparing pMBRT, FLASH, and combining FLASH-pMBRT exposures using appropriate tumor and normal-tissue models to clarify the interplay between spatially heterogeneous dose deposition and temporal ultra-high-dose-rate effects. Such studies could reveal unique biological responses distinct from either modality alone.

In summary, this study bridges the gap between theoretical proposals and experimental realization of FLASH-pMBRT, establishing the foundational framework for its future translation. With continued technical refinements—such as integration into conformal treatment geometries—and biological validation through in-vivo experiments, FLASH-pMBRT holds strong potential to become a next-generation radiotherapy modality offering unprecedented levels of precision and normal-tissue protection.

## 5. Conclusion

This study provides the first experimental validation of combining proton minibeam radiation therapy (pMBRT) with ultra-high-dose-rate (FLASH) delivery on a clinical proton therapy system. Using custom multi-slit collimators, we demonstrated submillimeter beam modulation under FLASH dose rates exceeding 100 Gy/s, achieving more than a 60-fold reduction in delivery time compared with conventional beams while maintaining consistent PVDR and spatial modulation quality.

The results confirm that FLASH-pMBRT is technically feasible with existing clinical infrastructure, laying the foundation for future conformal and biologically driven implementations. Ongoing work should focus on integrating this approach into spread-out Bragg peak (SOBP) delivery and conducting preclinical animal studies to assess potential synergistic biological effects arising from the combination of spatial and temporal dose-sparing mechanisms.

Together, these findings mark a critical step toward the clinical translation of FLASH-pMBRT, a next-generation radiotherapy paradigm with the potential to enhance tumor control while substantially reducing normal-tissue toxicity.

## Figures and Tables

**Table 1:** PVDR and dose-rate comparison between conventional and FLASH proton beams for 6.5 cm and 10 cm multi-slit collimators.

	Collimator Thickness		Depth 2 cm	Depth 4 cm	Depth 6 cm	Delivery Time
Conventional 226 MeV	6.5 cm	PVDR	2.66 ± 0.07	2.8 ± 0.15	2.16 ± 0.13	166 seconds
		PDR	0.2 Gy/s	0.1 Gy/s	0.1 Gy/s	
	10 cm	PVDR	4.64 ± 0.21	3.81 ± 0.15	2.40 ± 0.06	
		PDR	0.1 Gy/s	0.1 Gy/s	0.1 Gy/s	
FLASH 228MeV	6.5 cm	PVDR	2.57 ± 0.15	2.64 ± 0.13	2.20 ± 0.05	2.5 seconds
		PDR	12.2 Gy/s	9.9 Gy/s	7.9 Gy/s	
	10 cm	PVDR	4.37 ± 0.15	3.40 ± 0.12	2.55 ± 0.02	
		PDR	6.8 Gy/s	5.8 Gy/s	4.7 Gy/s	
		PDR_PBS	143.2 Gy/s	131.3 Gy/s	107.2 Gy/s	

## Data Availability

The dosimetry data presented in this study are available on request from the corresponding author; the patient data are not available to share due to patient privacy.

## Data Availability

The dosimetry data presented in this study are available on request from the corresponding author; the patient data are not available to share due to patient privacy.
